# Increased Risk of Cutaneous Diseases in Multiple Myeloma Patients

**DOI:** 10.7759/cureus.10356

**Published:** 2020-09-10

**Authors:** Talayesa Buntinx-Krieg, Ryan M McKee, Dawn Z Eichenfield, Amanda Marsch

**Affiliations:** 1 Department of Dermatology, University of California San Diego, La Jolla, USA

**Keywords:** multiple myeloma, myeloma of undetermined significance, skin conditions, oncology, monoclonal gammopathy

## Abstract

Introduction

Multiple myeloma (MM) is a monoclonal gammopathy characterized by malignant proliferation of plasma cells in the bone marrow, leading to the overproduction of monoclonal immunoglobulins. Knowledge of cutaneous findings associated with multiple myeloma is limited. This study aims to characterize cutaneous manifestations in patients with MM or monoclonal gammopathy of undetermined significance (MGUS).

Methods

This is a retrospective study of patients seen at a single institution between January 2000 and January 2019 with a diagnosis of “multiple myeloma,” “monoclonal gammopathy of undetermined significance,” or “smoldering myeloma,” and an on-site dermatology clinic visit.

Results

Twenty patients met the inclusion criteria. Most patients were male and Caucasian. Comorbid cutaneous malignancies were noted in 65% of patients (n = 13). Basal cell carcinoma (BCC) was characterized in 55% of patients (n = 11), followed by squamous cell carcinoma in 50% of patients (n = 10), and melanoma in 10% of patients (n = 2).

Conclusions

Patients with monoclonal gammopathy may be predisposed to developing cutaneous malignancies and skin infections. Given the low prevalence of monoclonal gammopathy, larger multi-center studies with a control cohort may be necessary to delineate the significance of these comorbid skin conditions.

## Introduction

Although multiple myeloma (MM) is an uncommon disease with a lifetime risk of 1 in 132 in the United States [1], recent advances in MM treatment have prolonged survival and led to an increased disease prevalence. Cutaneous involvement in MM most commonly results from metastasis and direct extension of disease from nearby tumor foci, presenting as palpable violaceous nodules on the trunk and extremities [2]. A recent Korean case series of 1,228 patients reported direct cutaneous involvement in only 1.14% of patients with MM. Direct cutaneous involvement was associated with significantly reduced overall survival in patients with MM [2]. Skin diseases associated with MM have been classified as specific and non-specific. The term “monoclonal gammopathy of cutaneous significance” was coined to describe dermatologic conditions strongly associated with a monoclonal gammopathy. Some examples of monoclonal gammopathy of cutaneous significance include purpura, hemorrhagic bullae, and macroglossia [3]. In contrast, the non-specific cutaneous findings associated with MM have not been well-established. One group did recently report a case series of four patients presenting with cutaneous manifestations such as leukocytoclastic vasculitis, pyoderma gangrenosum, and vesiculobullous disorders who were subsequently diagnosed with MM [4]. More recently, the International Myeloma Working Group has identified an increased risk of second primary malignancies in MM patients [5]. As such, a better understanding of cutaneous manifestations associated with MM could aid in earlier diagnosis of MM and be of prognostic significance. Here, we provide a retrospective review of patients with monoclonal gammopathy and analyze their associated cutaneous manifestations.

## Materials and methods

This is a retrospective analysis of patients diagnosed with monoclonal gammopathy between January 2000 and January 2019 at a single academic institution. Patients were identified using the following diagnoses: “multiple myeloma,” “monoclonal gammopathy of undetermined significance,” or “smoldering myeloma,” ICD-10 codes: C90.01, C90.00, D47.2 and ICD-9 codes: 203.0, 273.1. All patients ages 18-100 years who had visit(s) to an on-site dermatology clinic between January 2000 and January 2019 were included in the analysis. Twenty patients met the inclusion criteria. Chart review took place between November 1, 2019 and January 31, 2020, and de-identified data were imported into Microsoft Excel for analysis.

## Results

The majority of patients included in the analysis were male (12/20) and Caucasian (16/20). Thirteen patients were diagnosed with MM while seven patients had a diagnosis of MGUS. Patient characteristics are outlined in Table 1. As expected in an older cohort, most patients had also received a diagnosis of skin cancer (13/20) (Table 2). Basal cell carcinoma (BCC) was seen in 11/20 patients, while cutaneous squamous cell carcinoma and melanoma were diagnosed in 10/20 and 2/20 patients, respectively (Table 2). Several patients (8/20) had been diagnosed with both BCC and squamous cell carcinoma (SCC). Interestingly, two of three patients in our cohort with graft-versus-host disease (GVHD) eventually developed skin cancer, specifically BCC; however, none of the GVHD patients developed SCC.

A high number of infections were also observed in this cohort (17/20), which included skin infections (n = 17), urinary tract infections (n = 15), bacteremia (n = 13), upper respiratory tract infections (n = 8), and systemic viral infections (n = 4; Table 1). A number of other cutaneous conditions were observed in a minority of patients, which have been reported to aid in future studies (Table 1). Most patients received treatment for their plasma cell dyscrasia (13/20), which included allogeneic and autologous bone marrow transplantation (n = 8), lenalidomide (n = 12), dexamethasone (n = 10), bortezomib (n = 9), thalidomide (n = 6), and rituximab (n = 2; Table 1). Regarding treatment complications, drug-induced pancytopenia was most commonly reported (n = 7), followed by drug-induced neuropathy (n = 5), then GVHD (n = 3), and adrenal insufficiency (n = 3; Table 1).

 

**Table 1:** MM patient characteristics SD: standard deviation, BAPoma: BRCA1-associated-protein-oma, MM: multiple myeloma, MGUS: monoclonal gammopathy of undetermined significance, GVHD: graft-versus-host disease.

Characteristics			Value (%)
Patient characteristics			
	Number		20
	Mean age ± standard deviation, years		73.2 ± 14.99
	Age range, years		35-96
	Mean age at myeloma diagnosis ± standard deviation, years		67.8 ± 13.20
	Age range at myeloma diagnosis, years		51-90
	Male		12 (60)
	Female		8 (40)
	Ethnicity		
	Caucasian		16 (80)
	Hispanic		3 (15)
	Other		1 (5)
Myeloma characteristics			
	Diagnosis		
	MM		13 (65)
	MGUS		7 (35)
	Immunoglobulin subtype		
	IgA kappa		5 (25)
	IgG kappa		8 (40)
	IgG lambda		4 (20)
	IgD kappa		1 (5)
	Unknown		2 (10)
Other cancer diagnoses			
	Prostate cancer		3 (15)
	Lung cancer		1 (5)
	Bladder cancer		1 (5)
	Renal cell carcinoma		1 (5)
	Endometrial cancer		1 (5)
	Acute myeloid leukemia		1 (5)
	Mycosis fungoides		1 (5)
	Gallbladder cancer		1 (5)
Benign tumors			
	Benign sebaceous tumors		2 (10)
	Acantholytic acanthoma		1 (5)
	BRCA1-associated-protein-oma		1 (5)
Cutaneous diseases			
	Dermatomyositis		2 (10)
	Leukocytic vasculitis		1 (5)
	Mycosis fungoides (folliculotropic)		1 (5)
	Neutrophilic urticarial dermatosis		1 (5)
	Psoriasis		1 (5)
	Stasis dermatitis		2 (10)
	Stasis ulcer		2 (10)
	Porokeratosis		1 (5)
	Folliculitis		1 (5)
	Grover’s disease		1 (5)
	Chronic GVHD		2 (10)
	Acute GVHD		3 (15)
	Vulvar dysplasia		2 (10)
	Cellulitis		1 (5)
	Seborrheic dermatitis		1 (5)
	Xerosis		3 (15)
	Hypertrophic scars		1 (5)
	Lymphedema		1 (5)
	Lipodermatosclerosis		1 (5)
Skin infections			
	Escherichia coli		2 (10)
	Staphylococcus lugdunensis		1 (5)
	Methicillin-resistant Staphylococcus aureus		4 (20)
	Pseudomonas aeruginosa		3 (15)
	Propionibacterium acnes		1 (5)
	Dermabacter hominis		1 (5)
	Herpes simplex virus		3 (15)
	Herpes zoster		1 (5)
	Unknown		1 (5)
Urinary tract infections			
	E. coli		6 (30)
	P. aeruginosa		2 (10)
	Citrobacter koseri		2 (10)
	Proteus mirabilis		3 (15)
	Klebsiella		2 (10)
Bacteremia			
	E. coli		4 (20)
	Methicillin-sensitive S. aureus		4 (20)
	Citrobacter		1 (5)
	Klebsiella		1 (5)
	Methicillin-resistant S. aureus		1 (5)
	P. aeruginosa		1 (5)
	Mycobacterium kansasii		1 (5)
Upper respiratory tract infections			
	Candida albicans		2 (10)
	P. aeruginosa		2 (10)
	Streptococcus		1 (5)
	Aspergillus		1 (5)
	Klebsiella		2 (10)
Viral infections			
	Disseminated herpes simplex virus		1 (5)
	Human herpes virus-6 reactivation		1 (5)
	Epstein Barr virus reactivation		1 (5)
	Disseminated cytomegalovirus		1 (5)
Myeloma treatment medications			
	Ixazomib		3 (15)
	Dexamethasone		10 (50)
	Lenalidomide		12 (60)
	Bortezomib		9 (45)
	Anakinra		1 (5)
	Rituximab		2 (10)
	Pomalidomide		5 (25)
	Carfilzomib		2 (10)
	Cyclophosphamide		1 (5)
	Elotuzumab		2 (10)
	Thalidomide		6 (30)
	Biaxin		1 (5)
	Vincristine		1 (5)
	Doxorubicin		1 (5)
	Bone marrow transplant - autologous		5 (25)
	Bone marrow transplant - allogeneic		3 (15)
Myeloma treatment complications			
	Adrenal insufficiency		3 (15)
	GVHD		3 (15)
	Drug-induced neuropathy		5 (25)
	Drug-induced pancytopenia		7 (35)
	Drug-induced rash		1 (5)
	Acute myeloid leukemia		1 (5)
	Drug-induced pneumonitis		1 (5)

**Table 2:** Skin cancers in patients with MM MM: multiple myeloma, BCC: basal cell carcinoma, SCC: squamous cell carcinoma.

Skin Cancer Characteristics			Value (%)
Number of patients			13 (65)
	BCC		11 (55)
	Total number of lesions		26
	Mean number of lesions per patient		4.5
	Subtype prevalence (number of lesions)		
	Nodular		9
	Superficial		6
	Basosquamous		1
	Pigmented		4
	Micronodular		3
	Unknown		3
	SCC		10 (50)
	Total number of lesions		56
	Mean number of lesions per patient		5.5
	Subtype prevalence (number of lesions)		
	Bowen’s disease		30
	Invasive squamous cell carcinoma		22
	Keratoacanthoma		4
	Melanoma		2 (10)
	Total number of lesions		2
	Subtype prevalence (number of lesions)		
	Lentigo maligna		1
	Nodular		1

## Discussion

Although our cohort was smaller than some previously published studies, our results recapitulated those of prior larger cohort studies [2]. For example, similar to previous studies, our analysis also found IgG as the most common heavy chain subtype, followed by IgA [2]; however, in contrast to prior studies where the most commonly reported light chain was lambda, the most common light chain in our population was kappa.

While it may be difficult to draw definitive conclusions given our small patient population and lack of a control group, comorbid cutaneous malignancies (predominantly non-melanoma skin cancer) and infections appeared to be highly prevalent in this cohort. Caucasians have been reported to have a higher incidence of non-melanoma skin cancer than African Americans, while African Americans carry a twofold to threefold higher rate of MM than Caucasians [6]. Since 80% of our cohort was Caucasian, the higher rate of skin cancer noted here may not be applicable to a predominantly African American cohort. Therefore, it would be interesting to investigate the rates of skin cancer in a larger cohort with a higher percentage of African American patients.

Although more patients were diagnosed with BCC than SCC, SCC (30 Bowen’s disease lesions and 22 invasive SCC lesions) was the most commonly observed non-melanoma skin cancer in our cohort (56 SCCs compared to 26 BCCs total, Table 2). We suspect these findings are due to the impaired immune function inherent in MM patients, and may also be sequelae of MM therapies. These results are similar to those published in a recent large retrospective cohort study of 205 MM patients and 193 controls, which also demonstrated an increased incidence of skin cancer in MM patients (26.8% vs. 16.1% in controls; P = 0.009) [7]. Furthermore, immune dysfunction may also explain the high number of infections observed in our study; however, a larger retrospective cohort study with controls is needed to definitively ascertain whether the presence of monoclonal gammopathy predisposes patients to more infections.

There are several limitations of our study, which include a small patient population at a single academic institution, the retrospective nature of chart review, a lack of a control population, and a dependence on electronic medical record coding to identify patients. Unfortunately, by including only patients seen by dermatology, a majority of patients with plasma cell dyscrasias were excluded from this study. This finding in itself highlights that patients with monoclonal gammopathy may not be obtaining the necessary cutaneous care that should be a part of their management regimen, given their high prevalence of bone marrow transplant and immunosuppression. Despite these limitations, our findings are similar to those previously published in larger retrospective studies and also add to the literature by providing new insight into the significance of infections in MM and MGUS patients.

## Conclusions

MM is a rare disease characterized by the overproduction of monoclonal immunoglobulin. Despite being associated with significantly reduced overall survival, the cutaneous manifestations seen in patients with MM have not been thoroughly investigated. In this single institution retrospective review, we report a variety of comorbid skin findings in our cohort of patients with MM and MGUS. Of these, non-melanoma skin cancer and infections are especially prevalent, which may result from impaired immune defense in this patient population. Future studies are needed to further elucidate the diagnostic and prognostic significance of individual comorbid skin conditions in MM patients.
